# Early‐Onset Preeclampsia‐Derived hUC‐MSCs Exhibit Accelerated Initial Culture Kinetics Without Sustained Growth Advantage: A Comparative In Vitro Study

**DOI:** 10.1155/sci/3980640

**Published:** 2026-09-12

**Authors:** İbrahim Alptekin, Bilge Alptekin, Ezel Erkan, Ferda Topal Celikkan, Derya Uyan Hendem, Dilek Şahin, Alp Can

**Affiliations:** ^1^ Department of Histology and Embryology, Ankara University School of Medicine, Ankara, Türkiye, ankara.edu.tr; ^2^ The Graduate School of Health Sciences of Ankara University, Ankara, Türkiye; ^3^ Private Practice, Perinatology and Obstetrics and Gynecology, Diyarbakir, Türkiye; ^4^ Ankara Bilkent City Hospital, Ankara, Türkiye

**Keywords:** cell culture, early-onset preeclampsia (EOPE), human umbilical cord-derived mesenchymal stromal cells (hUC-MSCs), late-onset preeclampsia (LOPE), population doubling time

## Abstract

Preeclampsia (PE) is a pregnancy‐related disorder associated with significant maternal and fetal morbidity, and intrauterine conditions may influence the biological behavior of human umbilical cord (UC)‐derived mesenchymal stem cells (hUC‐MSCs). However, comparative data between early‐onset PE (EOPE) and late‐onset PE (LOPE) remain limited. This study aimed to compare the in vitro growth kinetics of hUC‐MSCs derived from pregnancies complicated by EOPE and LOPE. In this comparative in vitro study, UC samples (*n* = 66) were classified into three groups according to the maternal clinical condition: control (*n* = 22), EOPE (*n* = 22), and LOPE (*n* = 22). Cells were isolated using a mechanical explant method and cultured under standard conditions. Culture parameters, including cell‐outgrowth time, time to 90% confluency (*P*_0_), first passage duration (*P*_1_), and total culture duration (*P*_0_+*P*_1_), were recorded. Population doubling time (PDT) was calculated using both outgrowth‐adjusted and *P*_1_‐based approaches, and phenotypic characterization was performed by flow cytometry. Cell‐outgrowth time did not differ significantly among the groups (*p* = 0.930). *P*_0_ duration differed significantly among the groups (*p* < 0.001). EOPE‐derived cells exhibited a shorter *P*_0_ duration (19.00 ± 2.19 days) than both control‐derived cells (21.65 ± 1.63 days; Tukey‐adjusted *p* = 0.0004) and LOPE‐derived cells (20.71 ± 2.35 days; Tukey‐adjusted *p* = 0.0266). *P*_1_ duration and total culture duration did not differ significantly among the groups (*p* = 1.000 and *p* = 0.090, respectively). The outgrowth‐adjusted group‐level PDT estimate was lower in EOPE (16.6 h) than in LOPE (18.4 h) and controls (19.1 h), whereas the *P*_1_‐based PDT estimates were closely similar across the groups (~32 h). Total cell yield and viability were numerically similar across the groups. These findings indicate that EOPE‐derived hUC‐MSCs exhibit an early culture‐specific kinetic difference, predominantly reflected by a shorter *P*_0_ duration, without a sustained difference in subsequent *P*_1_ expansion or total culture duration. The accelerated early growth may reflect adaptive responses to the intrauterine environment rather than enhanced functional potential, suggesting that early culture parameters may serve as sensitive indicators of disease‐related alterations.

## 1. Introduction

Preeclampsia (PE) is a hypertensive disorder developing after 20 weeks of gestation, affecting 2%–8% of pregnancies worldwide and representing a major cause of maternal and fetal morbidity and mortality [1]. The etiology of PE is attributed to defects in early placental development, including inadequate trophoblast invasion, impaired spiral artery remodeling into high‐capacity, low‐impedance vessels, and abnormalities in chorionic villous development [2]. Based on the time of onset, PE can be classified into early‐onset PE (EOPE, 24–34 weeks) and late‐onset PE (LOPE, >34 weeks) forms, which differ in their underlying pathophysiology, clinical progression, and maternal and neonatal outcomes [3]. EOPE is characterized by abnormal placentation, impaired trophoblast invasion, and uteroplacental insufficiency, whereas LOPE is predominantly related to maternal cardiovascular factors, systemic inflammation, and metabolic predisposition [4, 5].

Human umbilical cord (UC)‐derived mesenchymal stem cells (hUC‐MSCs) are multipotent stromal cells characterized by high proliferative capacity, low immunogenicity, and strong immunomodulatory properties [6]. Due to these features and being obtained noninvasively from discarded perinatal tissue, hUC‐MSCs have been widely administered in regenerative medicine and therapeutic approaches in many clinical states. Importantly, accumulating evidence suggests that the biological characteristics of these cells are strongly influenced by intrauterine environmental conditions [7, 8].

In PE, placental hypoxia, oxidative stress, and chronic inflammation create a hostile intrauterine microenvironment that may impair hUC‐MSCs biology [9, 10]. Consistent with this, MSCs from PE and other complicated pregnancies exhibit reduced proliferative capacity, increased cellular senescence, and altered growth kinetics, suggesting that disease‐related intrauterine conditions may influence stem cell proliferation, differentiation, and functional properties [1, 10]. Despite the growing interest in this field, comparative data on the biological characteristics of hUC‐MSCs between EOPE and LOPE remain limited. Given the well‐established differences in placental pathology and disease severity between EOPE and LOPE, it is plausible that stem cell proliferation dynamics may also differ between these subtypes.

In vitro culture parameters, including the primary culture duration (*P*_0_), first passage duration (*P*_1_), and total culture duration, are common indicators of cell proliferation capacity and adaptability. The evaluation of these parameters may offer indirect yet valuable insights into the intrinsic cellular differences associated with pathological conditions. Therefore, the objective of this study was to compare the culture durations and in vitro growth kinetics of hUC‐MSCs derived from control, EOPE, and LOPE pregnancies.

## 2. Materials and Methods

### 2.1. Patient Selection

Participants were recruited from the Department of Obstetrics and Gynecology between April 2022 and April 2024. Informed consent was obtained from all participants prior to UC collection. The study protocol was approved by the Ethics Committee of the University (Protocol Number I04‐226‐22) and conducted in accordance with the Declaration of Helsinki. Participants were divided into three groups: Group 1 (control group) (*n* = 22); UC from healthy pregnancies delivered after 37 weeks of gestation; Group 2 (*n* = 22); UCs from pregnancies diagnosed with EOPE between 24 and 34 weeks, according to the International Society for the Study of Hypertension in Pregnancy (ISSHP) criteria; and Group 3 (*n* = 22); UCs from pregnancies with LOPE diagnosed after 34 weeks, also according to ISSHP criteria.

Exclusion criteria included a history of prepregnancy hypertension or the use of antihypertensive medications, as well as pre‐existing asthma, chronic obstructive pulmonary disease, diabetes mellitus, or cardiovascular disorders.

### 2.2. UC Processing and Primary Culture Establishment

**Figure 1:**
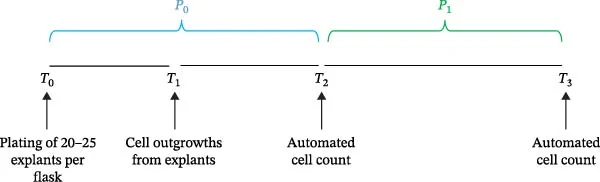
Schematic representation of the explant‐based culture timeline of hUC‐MSCs. The timeline illustrates the sequential stages of culture from primary explant (*P*_0_) to first passage (*P*_1_), including tissue processing, explant seeding, cell outgrowth, and subculturing. The experimental procedures corresponding to each stage are indicated.

UCs were obtained from cesarean deliveries. Samples were transported to the cell culture laboratory under sterile conditions in 150 mL of Leibovitz’s L‐15 medium. Upon arrival, necrotic and blood‐contaminated regions were removed, and tissues were washed with sterile phosphate‐buffered saline (PBS). Cord segments (5–7 cm) were prepared, and the umbilical arteries and vein were dissected and discarded. The remaining Wharton’s jelly was mechanically minced into ~1 mm^3^ fragments using sterile scissors. Stromal tissue fragments were centrifuged to eliminate debris, and the supernatant was discarded. The pellet was resuspended in Dulbecco’s modified Eagle medium/Ham’s F‐12 (DMEM/F‐12) supplemented with 10% fetal bovine serum (FBS), 1% antibiotic–antimycotic, 1% penicillin–streptomycin, and 1% L‐glutamine and seeded into T‐75 culture flasks at a density of 20–25 explants per flask. The plating of explants was defined as Time 0 (*T*_0_). Explants were incubated at 37°C in a humidified atmosphere with 5% CO_2_ and monitored every 24 h using an inverted microscope. The first appearance of adherent cells was recorded as *T*_1_ (cell outgrowths from explants). Cells were allowed to proliferate until reaching 80%–90% confluency, defined as *T*_2,_ when cells were detached and propagated onto a new flask as follows. The culture medium was removed, and cells were washed with Dulbecco’s PBS, followed by incubation with 5 mL of trypsin‐EDTA 0.05 % at 37°C until detachment. Trypsinization was neutralized by adding DMEM/F‐12 supplemented with 10% FBS, 1% antibiotic–antimycotic, 1% penicillin–streptomycin, and 1% L‐glutamine. A 1 mL aliquot was used for cell counting and viability assessment using a Vi‐CELL analyzer (Beckman Coulter). The remaining suspension was centrifuged at 200 g for 10 min. The supernatant was discarded, and the cell pellet was resuspended in a complete culture medium and seeded into new flasks at a density of 8300 cells/cm^2^. These cells were defined as passage 1 (*P*_1_) and monitored for 24 h intervals. The time at which *P*_1_ cells reached 90% confluency and then were counted was designated as *T*_3_. Therefore, the interval between *T*_0_ and *T*_2_ was designated as *P*_0_ and the interval between *T*_2_ and *T*_3_ as *P*_1_ (Figure 1). Representative images were acquired at each culture stage using an Olympus IX71 inverted microscope equipped with standard Hoffman modulation contrast optics and a 10× objective (Olympus, Tokyo, Japan), and image acquisition was performed using ToupView software.

### 2.3. Kinetics of Culture Duration

The primary culture kinetics of hUC‐MSCs were assessed by recording key temporal parameters during explant‐derived cell expansion. For each sample, the following variables were determined: cell‐outgrowth time, *P*_0_ duration, and *P*_1_ duration. Cell‐outgrowth time was defined as the interval between the initial plating of UC explants and the first observable outgrowth of adherent cells from the tissue fragments detected by microscopic examination. The *P*_0_ duration was defined as the interval from initial explant plating to the first passage (*T*_0_–*T*_2_), including the cell outgrowth period, whereas the *P*_1_ duration corresponded to the interval required for cells to reach 90% confluence following passaging (*T*_2_–*T*_3_). The total culture duration was calculated as the sum of the *P*_0_ and *P*_1_ durations (Figure 1). All intervals were recorded in days and expressed as mean ± standard deviation (mean ± SD) for each group (control, EOPE, and LOPE).

### 2.4. Population Doubling Time (PDT) Analysis

PDT was calculated using two different approaches. PDT was calculated using the following equation:

PDT=tlog2⁡NfNi,

where *t* represents the culture duration, *N*_*i*_ is the initial cell number, and *N*_*f*_ is the final cell number. In the cell outgrowth‐adjusted model, the time variable was defined as (*P*_0_+*P*_1_) − outgrowth time, and the initial cell number was assumed to be one, representing the first cell emerging from the explant. In the *P*_1_‐based model, the duration of *P*_1_ was used as the time variable, and the initial cell number was calculated based on the seeding density (8300 cells/cm^2^ × 75 cm^2^). In both models, the final cell number was represented by the corresponding group‐level mean harvested cell count. Accordingly, each model generated a single aggregated PDT value for each study group.

### 2.5. hUC‐MSC Validation

For phenotypic characterization, cryopreserved cells were thawed in a 37°C water bath and transferred into DMEM/Ham’s F‐12 culture medium supplemented with 10% FBS, 1% antibiotic–antimycotic solution, 1% penicillin–streptomycin, and 1% L‐glutamine. To remove residual dimethyl sulfoxide, the cell suspension was centrifuged at 200 × *g* for 10 min. The supernatant was discarded, and the cell pellet was resuspended in complete culture medium and cultured as passage 2 (*P*_2_). After subculturing to passage 3 (*P*_3_), cells were harvested and counted using a Vi‐CELL cell viability analyzer (Beckman Coulter).

For flow cytometric characterization, 1 × 10^6^ cells were resuspended in 200 µL PBS and stained using the Human Mesenchymal Stem Cell Analysis Kit (BD Biosciences; Catalog No. 562245). The antibody panel included the positive mesenchymal markers CD73, CD90, and CD105 and the negative hematopoietic‐lineage markers CD34, CD11b, CD19, CD45, and HLA‐DR. Twenty microliters of the antibody suspension were added to the cells, followed by incubation on ice for 20 min. Following incubation, the cells were centrifuged at 200 × g for 10 min. The supernatant was discarded, and the cell pellet was resuspended in 100 µL PBS. Marker expression was assessed using a BD Accuri C6 flow cytometer (BD Biosciences, USA), with 10,000 events acquired per sample. In the initial forward scatter/side scatter (FSC/SSC) plots, debris and subcellular particles were excluded, and the hUC‐MSC population was gated according to cell size and granularity. The background fluorescence was assessed using isotype‐matched control antibodies. The percentages of cells expressing the positive mesenchymal markers and negative hematopoietic‐lineage markers were determined relative to the corresponding isotype controls. Flow cytometry was performed to confirm the mesenchymal identity of the cultured cells and was not used as a comparative outcome among the study groups. A representative FSC/SSC gating plot used for flow cytometric analysis is provided in Figure S1.

### 2.6. Statistical Analysis

Statistical analyses were performed using the available individual sample‐level data for each outcome. The normality of continuous variables was assessed using the Shapiro–Wilk test, and the homogeneity of variances was evaluated using Levene’s test. Because cell‐outgrowth time did not meet the normality assumption, comparisons among the control, EOPE, and LOPE groups were performed using the Kruskal–Wallis test. *P*_0_ duration, *P*_1_ duration, and total culture duration met the assumptions for parametric analysis and were compared using one‐way analysis of variance. When the overall ANOVA result was statistically significant, Tukey’s honestly significant difference post hoc test was used for pairwise comparisons.

The *P*_0_, *P*_1_, and total culture duration analyses included 20 control, 21 EOPE, and 21 LOPE samples, whereas cell‐outgrowth time was available for 20 samples in each group because of technical exclusions during the initial phase of culture standardization and two missing cell‐outgrowth measurements. The aggregated PDT values and the descriptive total cell yield and viability data were not subjected to inferential statistical testing. All analyses were performed using IBM SPSS Statistics, Version 28.0. A two‐sided *p* value <0.05 was considered statistically significant.

Artificial intelligence tools (ChatGPT and OpenAI) were used solely for language editing of the manuscript. All scientific content, data analysis, and interpretations were performed and verified by the authors.

## 3. Results

### 3.1. Culture Kinetics of hUC‐MSCs

**Figure 2:**
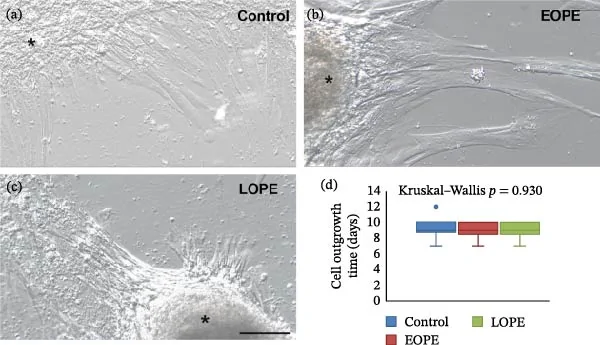
Cell outgrowth of hUC‐MSCs derived from control, EOPE, and LOPE groups. (a–c) Representative Hoffman modulation contrast micrographs demonstrating cell outgrowth from umbilical cord explants in control (a), EOPE (b), and LOPE (c) groups. Asterisks ( ^∗^) indicate explant regions. (d) Cell‐outgrowth time in each group (*n* = 20 per group). No statistically significant difference was detected among the groups (Kruskal–Wallis *H* = 0.146, *p* < 0.930). Scale bar: 50 µm.

**Figure 3:**
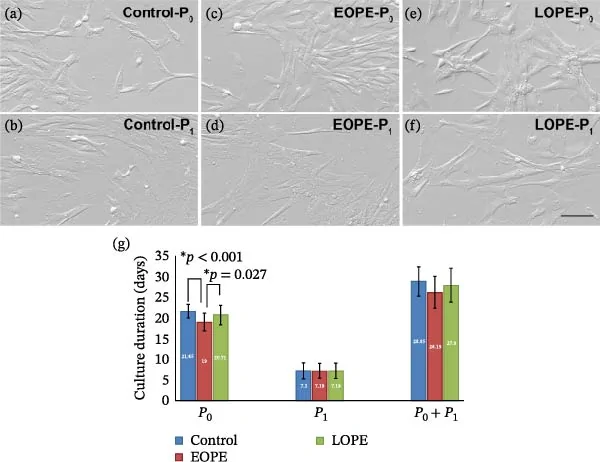
Early (*P*_0_ and *P*_1_) growth dynamics of hUC‐MSCs. (a, c, e) Representative Hoffman modulation contrast micrographs of *P*_0_ cultures and (b, d, f) representative micrographs of *P*_1_ cultures in the control, EOPE, and LOPE groups. *P*_0_ includes the initial cell outgrowth period. (g) *P*_0_, *P*_1_, and total culture duration in the control (*n* = 20), EOPE (*n* = 21), and LOPE (*n* = 21) groups. One‐way ANOVA followed by Tukey’s post hoc test showed that *P*_0_ duration was significantly shorter in the EOPE group than in the control group (adjusted *p* = 0.0004) and the LOPE group (adjusted *p* = 0.0266). No statistically significant group differences were observed for *P*_1_ duration or total culture duration. Scale bar: 50 µm. The asterisks ( ^∗^) indicate statistically significant pairwise differences in P0 duration following Tukey’s post hoc test.

Cell‐outgrowth time was available for 20 samples in each group. The median cell‐outgrowth time was 9.0 days (interquartile range, 8.75–10.0 days) in the control, EOPE, and LOPE groups. No statistically significant difference was observed among the groups (Kruskal–Wallis *H* = 0.146, *p* = 0.930) (Figure 2). *P*_0_ cells exhibited a smaller fibroblast‐like morphology, whereas *P*_1_ cells appeared relatively larger, consistent with previous reports [11]. The *P*_0_, *P*_1_, and total culture duration analyses included 20 control, 21 EOPE, and 21 LOPE samples. *P*_0_ duration differed significantly among the groups (one‐way ANOVA, *F* (2,59) = 8.546, *p* < 0.001). Tukey’s post hoc analysis showed that *P*_0_ duration was significantly shorter in the EOPE group (19.00 ± 2.19 days) than in both the control group (21.65 ± 1.63 days; adjusted *p* = 0.0004) and the LOPE group (20.71 ± 2.35 days; adjusted *p* = 0.0266). No statistically significant difference was observed between the control and LOPE groups (adjusted *p* = 0.330). *P*_1_ duration was similar among the control (7.20 ± 1.96 days), EOPE (7.19 ± 1.78 days), and LOPE (7.19 ± 1.83 days) groups (*p* = 1.000). Total culture duration was also not significantly different among the control (28.85 ± 3.56 days), EOPE (26.19 ± 3.88 days), and LOPE (27.90 ± 4.12 days) groups (one‐way ANOVA, *F* (2,59) = 2.505, *p* = 0.090) (Figure 3).

For the descriptive group‐level PDT calculation, the outgrowth‐adjusted effective proliferation time ([*P*_0_ + *P*_1_] − cell‐outgrowth time) was 19.6 ± 3.7 days in the control group, 16.7 ± 3.8 days in the EOPE group, and 18.4 ± 4.1 days in the LOPE group. PDT was estimated using two complementary calculation approaches to evaluate the proliferation kinetics of hUC‐MSCs.

**Figure 4:**
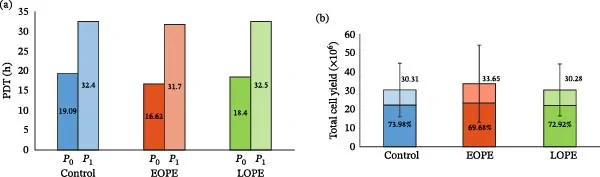
Group‐level population doubling time calculations and descriptive presentation of total cell yield and viability of hUC‐MSCs in the control, EOPE, and LOPE groups. (a) Group‐level PDT values calculated using the outgrowth‐adjusted and *P*_1_‐based models. The EOPE group showed a lower calculated PDT, particularly in the outgrowth‐adjusted model. Each model generated a single aggregated value per group; therefore, inferential statistical testing was not performed. (b) Descriptive presentation of total cell yield following two consecutive passages (*P*_0_ + *P*_1_). Total cell numbers were 30.31 × 10^6^, 33.65 × 10^6^, and 30.28 × 10^6^ cells for control, EOPE and LOPE groups, respectively. Dark‐colored bars represent viable cells, whereas light‐colored bars indicate nonviable cells. No inferential statistical comparisons were performed for the outcomes presented in this figure.

In the outgrowth‐adjusted model, based on the effective proliferative period defined as (*P*_0_+*P*_1_) − outgrowth time, the EOPE group demonstrated the shortest PDT (16.6 h), followed by the LOPE (18.4 h) and control groups (19.1 h) (Figure 4a). In the *P*_1_‐based model, which reflects post‐passage expansion under standard culture conditions, PDT values were higher and comparable across groups, with 31.7 h in the EOPE group, 32.5 h in the LOPE group, and 32.4 h in the controls. The group‐level PDT calculations showed a lower value in the EOPE group, particularly in the outgrowth‐adjusted model, whereas the *P*_1_‐based values were closely similar among the groups. Because each model generated a single aggregated PDT value for each group rather than individual sample‐level PDT measurements, inferential statistical comparisons could not be performed.

Total cell yield and viability were descriptively presented to document the culture output across the control, EOPE, and LOPE groups. Mean total cell yields were numerically similar, ranging from 30.28 × 10^6^ to 33.65 × 10^6^ cells, while mean viable cell numbers ranged from 22.08 × 10^6^ to 23.45 × 10^6^ cells. Mean viability rates were also within a similar numerical range from 69.7% to 73.9% (Figure 4b). No inferential statistical comparisons were performed for these outcomes.

### 3.2. In Vitro Characterization of hUC‐MSCs

**Figure 5:**
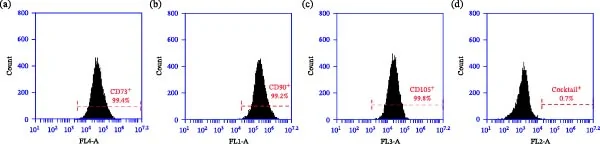
Immunophenotypic characterization of hUC‐MSCs by flow cytometry. Representative flow‐cytometry histograms of *P*_3_ hUC‐MSCs demonstrate high expression (>95%) of the mesenchymal markers (a) CD73, (b) CD90, and (c) CD105 and (d) the hematopoietic‐lineage marker cocktail comprising CD34, CD11b, CD19, CD45, and HLA‐DR. Flow cytometry was performed to confirm the mesenchymal identity of the cultured cells and was not used as a comparative outcome among the study groups.

*P*_3_ cells were analyzed by flow cytometry to confirm their mesenchymal immunophenotype. The positive mesenchymal markers CD73, CD90, and CD105 were expressed at levels exceeding 95%, whereas the hematopoietic‐lineage markers CD34, CD11b, CD19, CD45, and HLA‐DR showed expression levels below 1% (Figure 5).

## 4. Discussion

In this study, we investigated the culture kinetics and proliferation characteristics of hUC‐MSCs derived from EOPE and LOPE pregnancies compared with healthy controls using an explant‐based primary culture model. EOPE‐derived hUC‐MSCs exhibited a significantly shorter *P*_0_ duration than both control‐ and LOPE‐derived cells. In contrast, cell‐outgrowth time, *P*_1_ duration, and total culture duration did not differ significantly among the groups. The group‐level outgrowth‐adjusted PDT estimate was also numerically lower in the EOPE group, whereas *P*_1_‐based PDT estimates, total cell yield, and viability were numerically similar among the groups. These findings suggest that EOPE‐associated differences are predominantly expressed during the early primary culture phase and do not persist during subsequent *P*_1_ expansion [4, 12].

In the context of PE, the intrauterine environment is characterized by placental hypoxia, oxidative stress, and inflammatory signaling, which are known to influence the stem cell behavior. Hypoxia‐inducible pathways, particularly those mediated by hypoxia‐inducible factor (HIF)‐1α, have been shown to enhance MSC proliferation and survival under stress conditions [13]. Additionally, disease‐related microenvironmental stress may induce adaptive or preconditioned states in stem cells, leading to transient increases in proliferative activity once cells are established in culture [14]. Taken together, these findings suggest that while the initial release and activation of cells from the explant remain unaffected, PE‐associated conditions may selectively modulate downstream proliferative responses, resulting in accelerated expansion during the early culture phase. The absence of differences in the cell outgrowth period between groups further supports this interpretation. The outgrowth period reflects the latency period required for the first cells to emerge from the explant, and similar values across groups indicate that initial activation and migration processes are preserved. Therefore, the observed differences in *P*_0_ duration likely reflect alterations in the proliferative phase rather than the initiation of cell outgrowth.

Despite these early kinetic differences, the total cell yield and viability were numerically similar across the groups. This suggests that EOPE‐derived cells may exhibit transiently enhanced proliferation without a sustained increase in expansion capacity. Wagner et al. [11] demonstrated that MSCs undergo a continuous and organized process of replicative senescence, characterized by progressive changes in the proliferation rate, morphology, and cellular function across passages, indicating that proliferative behavior is dynamic rather than constant. Similarly, Phinney [15] highlighted the inherent functional heterogeneity of MSC populations, emphasizing that cells derived from different microenvironments may exhibit distinct proliferative and functional properties without necessarily affecting overall expansion outcomes. In this context, our findings suggest that EOPE‐associated conditions may transiently enhance early proliferative activity, while subsequent *P*_1_ expansion remained numerically similar among the groups.

To further characterize the proliferation dynamics, we applied two complementary group‐level PDT calculation models. The EOPE group showed numerically lower PDT estimates, with the reduction being more pronounced in the outgrowth‐adjusted model. In contrast, the *P*_1_‐based estimates were closely similar among the groups. Because these calculations generated a single aggregated PDT value for each group, they were interpreted descriptively rather than as inferential statistical comparisons. The outgrowth‐adjusted model showed the most pronounced numerical reduction, suggesting that EOPE‐associated effects may predominantly influence the active proliferation phase following cell outgrowth. In contrast, the *P*_1_‐based model demonstrated similar PDT values across groups, indicating that once cells adapt to standard in vitro conditions, proliferation kinetics become comparable. This convergence may reflect a selection process favoring more proliferative and adaptable subpopulations during culture expansion [11].

These observations can be interpreted in light of the intrauterine environment in PE cases. EOPE is associated with placental hypoxia, oxidative stress, and chronic inflammation, all of which can influence stem cell behavior [16, 17]. Hypoxic conditions, in particular, have been shown to enhance MSC proliferation via activation of HIF‐1α and downstream signaling pathways, including VEGF‐mediated mechanisms [13]. Thus, the shorter *P*_0_ duration and reduced PDT observed in EOPE‐derived cells may reflect an adaptive or preconditioned state induced by the pathological intrauterine environment.

Another factor to consider is the explant culture method used in this study. Compared to enzymatic digestion, explant‐based isolation allows for selective migration of adherent cell populations and may favor the expansion of more proliferative subpopulations [18]. This method may amplify early differences in cell behavior while contributing to the observed homogenization of proliferation kinetics in later passages [6]. Additionally, although samples were consistently prepared from Wharton’s jelly, subtle regional differences within the UC may influence cell characteristics [19, 20]; however, precise standardization of these subregions remains challenging in practice.

Several limitations should be acknowledged. First, PDT calculations were based on group‐level cell counts rather than individual sample‐based values, precluding statistical comparison of PDT between groups. Second, the use of theoretical initial cell numbers in certain models reflects a modeling approach rather than a direct biological measurement. An additional limitation is the inherent difference in gestational age among the study groups. EOPE and LOPE are clinically defined according to gestational age at disease onset, while the control group consisted of term pregnancies. Therefore, the effects of gestational maturity cannot be completely separated from those associated with the PE subtype in the present study design. Accordingly, the observed kinetic differences should be interpreted as being associated with the EOPE clinical phenotype rather than as evidence of an independent causal effect of disease onset timing. Future studies incorporating gestational age‐matched preterm control pregnancies will be required to distinguish the effects associated with PE from those related to gestational maturity. Another important limitation of this study is that although proliferation kinetics and expansion dynamics were comprehensively evaluated, no direct conclusions can be drawn regarding the functional capacity of hUC‐MSCs derived from PE pregnancies. While EOPE‐derived cells demonstrated a shorter *P*_0_ duration and a numerically lower outgrowth‐adjusted group‐level PDT estimate, indicating an apparent proliferative advantage during early culture stages, these findings reflect quantitative growth characteristics rather than qualitative cellular function. It is well established that the proliferation rate does not necessarily correlate with functional properties such as differentiation potential, immunomodulatory capacity, or paracrine activity [14]. Moreover, the observed convergence of proliferation kinetics during later passages suggests that cells may undergo adaptation to in vitro conditions over time; however, this numerical adaptation does not guarantee the restoration of functional competence. Therefore, future studies incorporating functional assays—such as differentiation capacity, cytokine secretion profiling, and immunomodulatory analyses—are required to determine whether PE‐associated alterations in early proliferation are accompanied by changes in the biological function of hUC‐MSCs.

## 5. Conclusion

In conclusion, hUC‐MSCs derived from EOPE pregnancies exhibited an early culture‐specific kinetic difference, reflected by a significantly shorter *P*_0_ duration than both control‐ and LOPE‐derived cells and a numerically lower outgrowth‐adjusted group‐level PDT estimate. However, cell‐outgrowth time, *P*_1_ duration, and total culture duration did not differ significantly among the groups, indicating that the early kinetic difference was not sustained during subsequent culture expansion.

## Author Contributions

Concept, design: İbrahim Alptekin and Alp Can. Materials: Derya Uyan Hendem and Dilek Şahin. Tissue processing: İbrahim Alptekin, Bilge Alptekin, Ezel Erkan, and Ferda Topal Celikkan. Data collection or processing, analysis or interpretation: İbrahim Alptekin. Literature search, writing: İbrahim Alptekin and Bilge Alptekin. Funding: Alp Can.

## Funding

This study was funded by the Ankara University Scientific Research Fund under Project Number TSG‐2022‐2545.

## Disclosure

This manuscript has not been previously published and is not under consideration for publication elsewhere.

## Ethics Statement

Ethics committee approval was obtained from The University Ethical Review Board in Human Research (21.04.2022‐İ04‐226‐22).

## Conflicts of Interest

The authors declare no conflicts of interest.

## Supporting Information

Additional supporting information can be found online in the Supporting Information section.

## Supporting information


**Supporting Information** Figure S1: Representative FSC/SSC gating plot used for flow‐cytometric analysis of hUC‐MSCs.

## Data Availability

The data that support the findings of this study are available from the corresponding author upon reasonable request.
