# Supplementary material for: Early‐Onset Preeclampsia‐Derived hUC‐MSCs Exhibit Accelerated Initial Culture Kinetics Without Sustained Growth Advantage: A Comparative In Vitro Study
Source: Stem Cells Int. 2026 Sep 12;2026:3980640. doi: 10.1155/sci/3980640 (PMC13570448; doi:10.1155/sci/3980640)
Supplement: Supplementary file 1 [file SCI-2026-3980640-s001.docx]

**Supplementary Figure S1. Representative FSC/SSC gating plot used for flow-cytometric analysis of hUC-MSCs.** Debris and subcellular particles located in the lower-left region of the FSC-A/SSC-A plot were excluded, and the main hUC-MSC population was gated according to cell size (forward scatter) and granularity (side scatter). In the representative plot shown, the gated cell population comprised 84.6% of acquired events.
